# Supplementary material for: Causal interplay between lactose intolerance and gut microbiota: a combined bidirectional Mendelian randomization and in vivo validation study
Source: Front Nutr. 2026 Jun 1;13:1803337. doi: 10.3389/fnut.2026.1803337 (PMC13265576; doi:10.3389/fnut.2026.1803337)
Supplement: Supplementary file 1 [file Data_Sheet_1.zip › supplementary materials/Forward/leave one out cross validation/ebi-a-GCST90027525.finngen_R12_E4_LACTONAS.pdf]

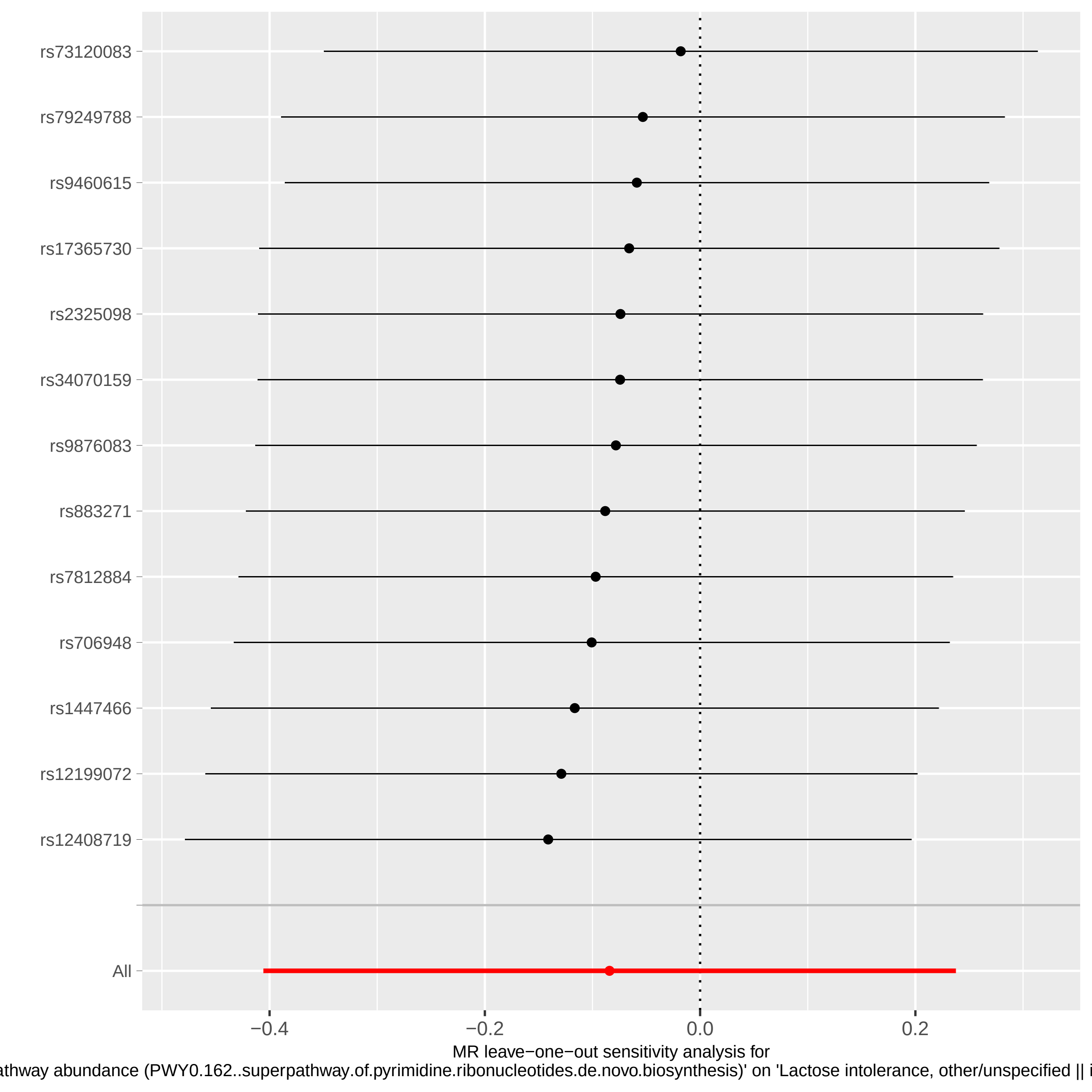

rs73120083
rs79249788
rs9460615
rs17365730
rs2325098
rs34070159
rs9876083
rs883271
rs7812884
rs706948
rs1447466
rs12199072
rs12408719
All
−0.4
−0.2
0.0
0.2
MR leave−one−out sensitivity analysis for
athway abundance (PWY0.162..superpathway.of.pyrimidine.ribonucleotides.de.novo.biosynthesis)' on 'Lactose intolerance, other/unspecified || i
